# CD59 signaling and membrane pores drive Syk-dependent erythrocyte necroptosis

**DOI:** 10.1038/cddis.2015.135

**Published:** 2015-05-28

**Authors:** T J LaRocca, E A Stivison, T Mal-Sarkar, T A Hooven, E A Hod, S L Spitalnik, A J Ratner

**Affiliations:** 1 Department of Pediatrics, Columbia University, New York, NY, USA; 2 Institute of Human Nutrition, Columbia University, New York, NY, USA; 3 Department of Pathology and Cell Biology, Columbia University, New York, NY, USA

## Abstract

Mature erythrocytes (red blood cells (RBCs)) undergo the programmed cell death (PCD) pathway of necroptosis in response to bacterial pore-forming toxins (PFTs) that target human CD59 (hCD59) but not hCD59-independent PFTs. Here, we investigate the biochemical mechanism of RBC necroptosis with a focus on the mechanism of induction and the minimal requirements for such RBC death. Binding or crosslinking of the hCD59 receptor led to Syk-dependent induction of vesiculated morphology (echinocytes) that was associated with phosphorylation of Band 3 and was required for Fas ligand (FasL) release. FasL-dependent phosphorylation of receptor-interacting protein kinase 1 (RIP1) in combination with plasma membrane pore formation was required for execution of RBC necroptosis. RIP1 phosphorylation led to the phosphorylation of RIP3, which was also critical for RBC necroptosis. Notably, RBC necroptosis was mediated by FasL and not by other candidate inducers, including tumor necrosis factor alpha (TNF-*α*) and TNF-related apoptosis-inducing ligand (TRAIL). Other types of RBC damage, such as eryptotic damage, failed to induce necroptosis when combined with hCD59 crosslinking. This work sheds light on the requirements for this recently discovered PCD in RBCs and provides a clear picture of the biochemical mechanism of induction of RBC necroptosis.

Recently, we demonstrated that bacterial pore-forming toxins (PFTs) that target human CD59 (hCD59) induce programmed necrosis (necroptosis) in primary erythrocytes (red blood cells, RBCs).^1^ This observation was striking, as RBC lack nuclei and mitochondria. Nonetheless, RBC necroptosis proceeded in a manner similar to that observed in nucleated cells, requiring Fas and Fas ligand (Fas/FasL), mixed lineage kinase domain-like protein, and the phosphorylation of receptor-interacting protein kinase 1 (RIP1) kinase.^1^ RBC necroptosis was antagonized by caspase-8 and was associated with necrosome formation and conserved necroptosis effector pathways including acid sphingomyelinase-dependent ceramide formation, NADPH oxidase/iron-dependent reactive oxygen species formation, and glycolytic formation of advanced glycation end products (AGEs). While RBC necroptosis shared many molecular steps with canonical necroptosis pathways, it was distinct from eryptosis, a RBC-specific programmed cell death (PCD) that functions as a trigger for RBC clearance by macrophages.^1, 2^

Proteins that induce plasma membrane pores are a common biological theme and may be pathogen produced, such as bacterial PFTs, or host produced, such as the membrane attack complex (MAC) of the complement system.^3, 4^ Pore formation may kill cells through osmotic lysis or may activate target cell signaling pathways including proinflammatory, membrane repair, and PCD modules.^4^ However, the specific mechanisms linking bacterial PFTs to RBC necroptosis have not been described.

In this study, we sought to understand the mechanism of induction of RBC necroptosis by bacterial PFTs and the minimal requirements for such death in these target cells. In particular, we show that hCD59 signaling, induced by receptor crosslinking, results in Syk-dependent phosphorylation of Band 3 leading to vesicle (echinocyte) formation and release of FasL. Released FasL induces the phosphorylation of RIP1, which in turn leads to RIP3 phosphorylation. The FasL-dependent phosphorylation of RIP1/RIP3 produces RBC death by necroptosis only when combined with functional pore formation. The ability for necroptosis to proceed in RBCs differs depends on the size and nature of membrane pores. Additionally, we show that RBC necroptosis is mediated only by FasL and not by other known necroptotic mediators/stimuli, including tumor necrosis factor alpha (TNF-*α*) and TNF-related apoptosis-inducing ligand (TRAIL).

## Results

### RBC necroptosis induced by hCD59-specific PFTs is mediated by FasL but not by other known mediators/stimuli of necroptosis

**Figure 1:**
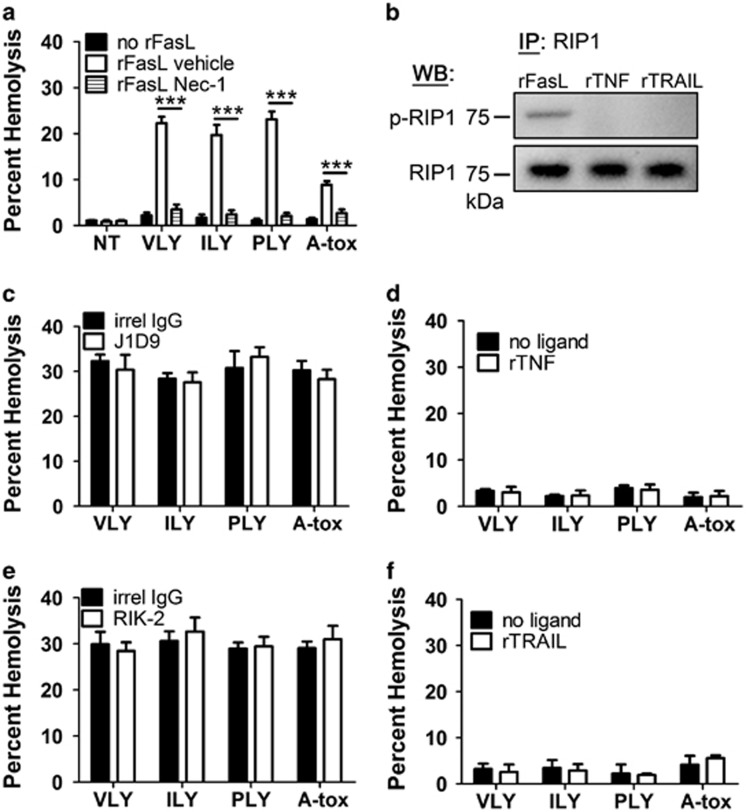
Necroptosis depends on FasL but not TNF-*α* or TRAIL in RBCs. (**a**) Addition of exogenous rFasL (10 ng/ml) with low doses of PFTs (0.02 HU) leads to necroptosis in RBCs that is prevented by the RIP1 inhibitor, necrostatin-1 (nec-1, 50 *μ*M). (**b**) RIP1 IPs showing phosphorylation of RIP1 (p-RIP1) in response to rFasL (1 *μ*g/ml) but not rTNF-*α* (10 *μ*g/ml) or rTRAIL (10 *μ*g/mL) following 1 h of exposure. (**c**) Neutralization of TNF-*α* with the mAb J1D9 (1 *μ*g/ml), (**d**) addition of rTNF-*α* (5 *μ*g/ml), (**e**) neutralization of TRAIL with the mAb RIK-2 (1 *μ*g/ml) or (**f**) addition of rTRAIL (5 *μ*g/ml) has no effect on RBC death by VLY, ILY, PLY or A-tox. Time points for all hemolysis assays were 30 min. Error bars in hemolysis assays represent standard deviation. All hemolysis assays are the results of three independent experiments. Two-way ANOVA with Bonferroni posttest, ***P<0.001

We previously demonstrated that RBC necroptosis, induced by the hCD59-specific PFTs vaginolysin (VLY) and intermedilysin (ILY), is dependent on Fas-FasL signaling, as a monoclonal antibody targeting FasL (mAb NOK-1) significantly inhibited RBC death.^1^ Recombinant FasL (rFasL) enhances RBC death by near sublytic quantities of PFTs in a necrostatin-1-inhibitable manner (Figure 1a). The hCD59-specific PFTs, VLY and ILY, induce FasL-dependent phosphorylation of RIP1 in RBCs,^1^ and addition of rFasL (but not TNF-*α* or TRAIL) to RBCs induces the phosphorylation of RIP1 (Figure 1b). Due to the fact that rFasL is sufficient to trigger RIP1 phosphorylation, its inclusion allows hCD59-independent PFTs, pneumolysin (PLY) and *Staphylococcus aureus* alpha toxin (A-tox), to induce necroptosis in RBCs (Figure 1a).^1^

As FasL triggers necroptosis in some nucleated cells,^5, 6, 7^ we asked whether two other defined stimuli of necroptosis, TNF-*α* and TRAIL,^6, 7^ have a role in this PCD in RBCs. In contrast to rFasL, addition of rTNF-*α* or rTRAIL to RBCs failed to induce RIP1 phosphorylation (Figure 1b). Additionally, while neutralization of FasL with a mAb inhibits RBC necroptosis by VLY and ILY,^1^ similar neutralization of TNF-*α* or TRAIL had no effect on necroptosis by these hCD59-specific PFTs (Figures 1c and e). Moreover, while the addition of exogenous rFasL endows PFTs with the ability to induce RBC necroptosis, regardless of intrinsic hCD59 specificity, similar addition of rTNF-*α* or rTRAIL had no effect on RBC death (Figures 1d and f). These results indicate that FasL is a critical mediator of RBC necroptosis.

### Binding or crosslinking of hCD59 leads to phosphorylation of RIP1 in human RBCs

**Figure 2:**
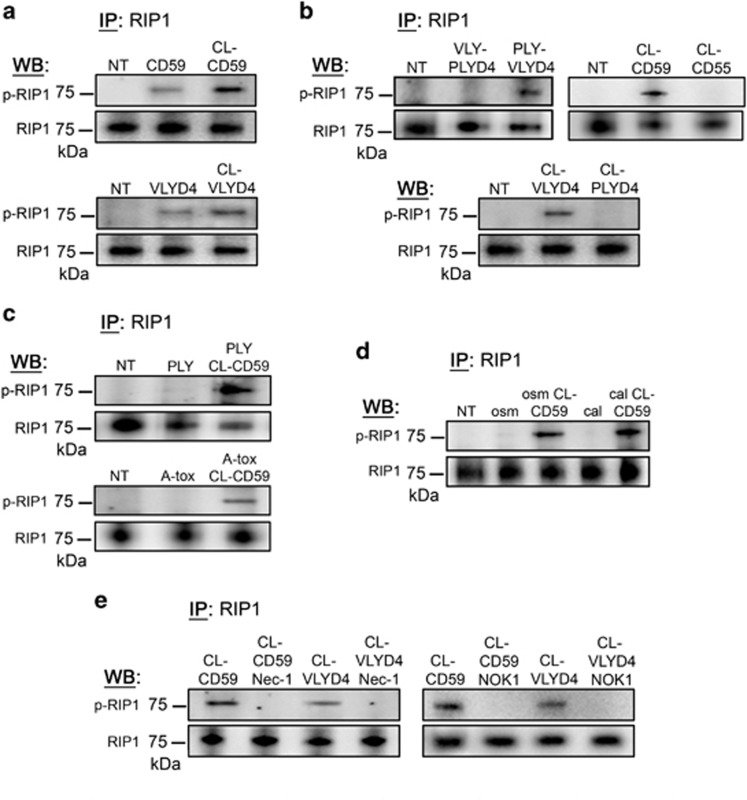
Binding or crosslinking of the hCD59 receptor leads to FasL-dependent phosphorylation of RIP1 in RBCs. (**a**) RIP1 IPs showing p-RIP1 in response to binding of hCD59 by the specific mAb MEM-43 (CD59, 1 *μ*g/mL) or a His-tagged version of the binding domain of VLY (VLYD4, 100 ng/ml). Further crosslinking of hCD59 with an anti-mouse IgG pAb (CL-CD59) or an anti-His mAb (CL-VLYD4) leads to more robust levels of p-RIP1. (**b**) IPs similar to those in (**a**) showing p-RIP1 specifically in response to hCD59 binding. A version of PLY modified with the binding domain of VLY (PLY-VLYD4) induces p-RIP1 while VLY modified with the binding domain of PLY (VLY-PLYD4) does not. RIP1 is phosphorylated specifically in response to CL-CD59 or CL-VLYD4. Crosslinking of hCD55 (CL-CD55) or PLYD4 (CL-PLYD4) does not result in p-RIP1. The p-RIP1 induced by CL-CD59 is retained in combination with (**c**) the hCD59-independent PFTs, PLY or A-tox, or (**d**) the eryptosis stimuli, hyperosmotic stress (osm) or excess calcium (cal). (**e**) The p-RIP1 induced by CL-CD59 or CL-VLYD4 is prevented by the RIP1 inhibitor nec-1 (50 *μ*M) or neutralization of FasL (NOK-1, 1 *μ*g/ml). Treatments for all experiments except for eryptosis were 1 h. Eryptosis stimuli were added to RBCs for a period of 24 h. When nec-1 or mAb NOK-1 was used, RBCs were treated with these inhibitors for 1 h before the addition of stimuli

hCD59-specific PFTs (VLY and ILY) induce RBC necroptosis while hCD59-independent PFTs do not,^1^ but it is not known whether hCD59 ligation itself induces necroptosis signaling pathways. Treatment of RBCs with either an hCD59-specific mAb or a histidine-tagged version of the VLY binding domain (VLYD4) triggered RIP1 phosphorylation in RBCs (Figure 2a). Cross-linking of the hCD59-binding reagents with an anti-mouse IgG pAb (for the mAb) or an anti-His mAb (for VLYD4) resulted in more robust RIP1 phosphorylation (Figure 2a). Thus, signaling through hCD59 is sufficient to induce RIP1 phosphorylation in human RBC.

The hCD59-specific PFT VLY induces RIP1 phosphorylation in RBCs, while the related but non-hCD59-specific PLY does not.^1^ To determine whether toxin-induced RIP1 phosphorylation depends entirely on PFT binding to hCD59, we generated binding domain-swapped mutant versions of these PFTs (Figure 2b). When VLY was modified with the binding domain of PLY (VLY-PLYD4), it did not induce RIP1 phosphorylation (Figure 2b). On the other hand, when PLY was modified with the binding domain of VLY (PLY-VLYD4) such that it was capable of binding hCD59, it did result in RIP1 phosphorylation (Figure 2b). The phosphorylation of RIP1 is specific to CD59 signaling in RBCs as crosslinking of a related membrane protein, CD55, did not result in RIP1 phosphorylation (Figure 2b). Likewise, when we crosslinked the binding domain of PLY (PLYD4), which does not bind hCD59, there was no phosphorylation of RIP1 (Figure 2b). These results demonstrate that PFT-mediated RIP1 phosphorylation depends on hCD59 ligation in RBCs.

The induction of RIP1 phosphorylation by mAb crosslinked hCD59 (CL-CD59) in RBCs was retained when combined with the hCD59-independent PFTs, PLY or A-tox (Figure 2c), or with two distinct inducers of eryptosis, hyperosmotic stress or excess calcium (Figure 2d). In addition, phosphorylation of RIP1 induced by hCD59 signaling via hCD59-specific mAb or VLYD4 was inhibited by both nec-1 and mAb against FasL (Figure 2e).

### Crosslinking of hCD59 as well as hCD59-specific PFTs induces the phosphorylation of RIP3, which is required for RBC necroptosis

**Figure 3:**
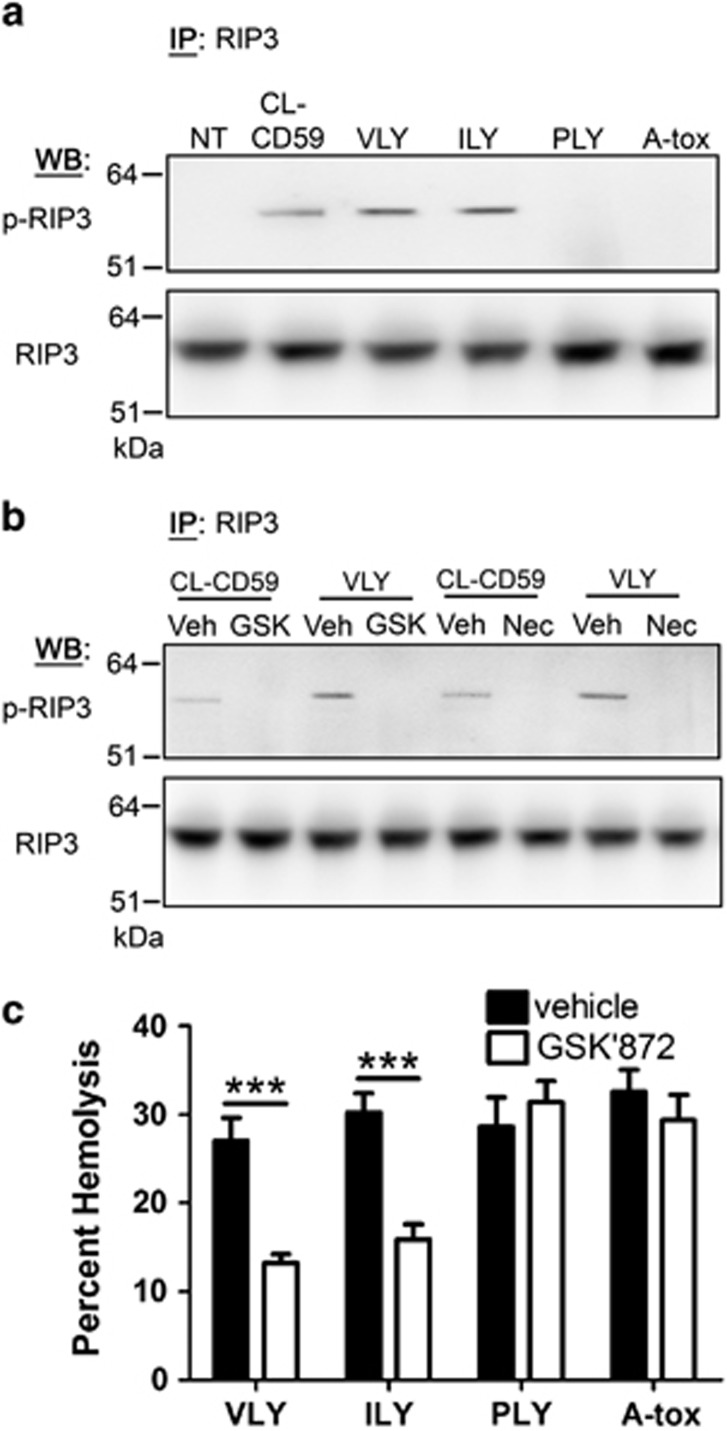
hCD59 signaling leads to RIP1-dependent phosphorylation of RIP3, which is critical for RBC necroptosis. (**a**) RIP3 IPs showing the phosphorylation of RIP3 (p-RIP3) in response to the hCD59-specific PFTs, VLY and ILY but not by the hCD59-independent PFTs, PLY and A-tox. Crosslinking of hCD59 with mAbs (CL-CD59) induces p-RIP3 in the absence of PFTs. (**b**) IPs showing that p-RIP3, induced by CL-CD59 or VLY, are prevented by inhibition of RIP3 with GSK'872 (2 *μ*M) or inhibition of RIP1 with nec-1 (50 *μ*M). (**c**) Hemolysis assay showing that inhibition of RIP3 with GSK'872 (2 *μ*M) reduces RBC death by VLY and ILY but not by PLY or A-tox. Treatment times for IPs were 1 h. Time points for hemolysis assays were 30 min following 1 h incubation with GSK'872. Error bars in the hemolysis assay represent standard deviation. The hemolysis assay is the results of three experiments

Similar to the phosphorylation of RIP1 (Figure 2),^1^ RIP3, which is also critical for necroptosis,^7^ is phosphorylated in response to crosslinking of hCD59 or hCD59-specific PFTs, VLY and ILY in RBCs (Figure 3a). This is consistent with necrosome formation in RBC necroptosis.^1^ hCD59-independent PFTs, PLY and A-tox, did not induce RIP3 phosphorylation. The phosphorylation of RIP3 was prevented by the recently characterized inhibitor of RIP3, GSK'872,^8^ or by RIP1 inhibition with nec-1 (Figure 3b). That nec-1 prevents RIP3 phosphorylation indicates that this kinase is phosphorylated due to the action of RIP1 as is the case in nucleated cells.^7^ Inhibition of RIP3 with GSK'872 also reduced RBC death by VLY and ILY but not by the hCD59-independent PFTs, PLY and A-tox, demonstrating a critical role for RIP3 in the necroptotic death of RBCs (Figure 3c).

### Binding or crosslinking of hCD59 leads to necroptosis of RBCs when combined with PLY

**Figure 4:**
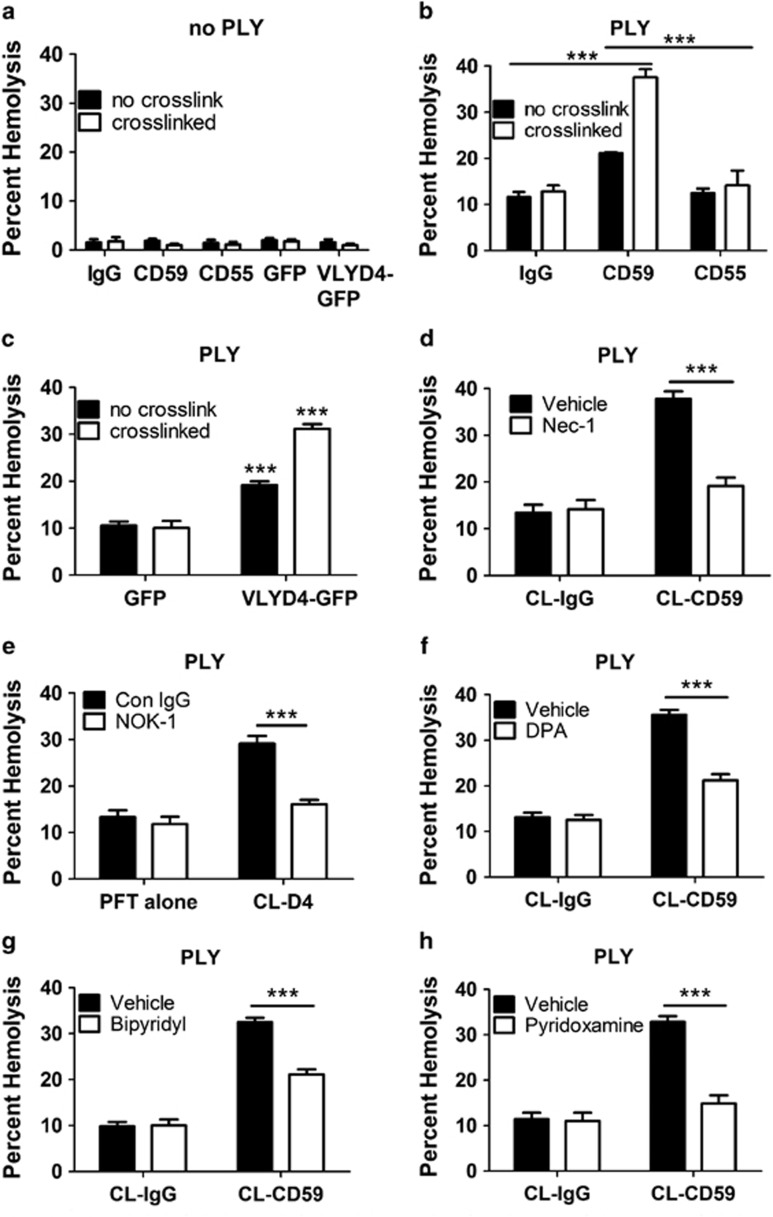
The combination of hCD59 crosslinking and PLY induces RBC necroptosis. (**a**) Hemolysis assay showing that mAb binding or crosslinking of hCD59 (CD59) or hCD59 binding or crosslinking with the binding domain of VLY fused to GFP (VLYD4-GFP) does not induce death of RBCs. When hCD59 is bound or crosslinked with (**b**) mAb against hCD59 or (**c**) VLYD4-GFP it results in an enhancement of RBC death by the hCD59-independent PLY (0.2 HU). Enhanced RBC death by PLY in the presence of crosslinked hCD59 is prevented by (**d**) the RIP1 inhibitor, nec-1 (50 *μ*M) or (**e**) the FasL neutralizing mAb, NOK-1 (1 *μ*g/ml). Enhanced RBC death by PLY in the presence of crosslinked hCD59 is reduced by inhibitors of downstream effector pathways in RBC necroptosis including (**f**) inhibition of ceramide formation with acid sphingomyelinase (aSMase) inhibitor desipramine (DPA, 20 *μ*M), (**g**) inhibition of reactive oxygen species (ROS) with the iron chelator 2,2 bipyridyl (bipyridyl, 100 *μ*M) or (**h**) inhibition of AGEs with pyridoxamine (1 mM). Time points for hemolysis assays were 30 min. When pharmacological or mAb inhibitors were used RBCs were treated for 1 h before the addition of stimuli. IgG=irrelevant IgG control, CD55=mAb against hCD55, GFP=addition of rGFP alone. Error bars in hemolysis assays represent standard deviation. All hemolysis assays are the results of three independent experiments. Two-way ANOVA with Bonferroni posttest, ***P<0.001

Although crosslinking of hCD59 with antibody or VLYD4 induces RIP1 phosphorylation, it is not sufficient to induce necroptotic death in RBCs (Figure 4a). However, similar to the effect of rFasL, hCD59 ligation enhances RBC death by hCD59-independent PLY (Figures 4b and c). This suggests that, at minimum, RBC necroptosis requires hCD59 signaling and a PFT. RBC death by necroptosis induced by PLY combined with CL-CD59 is prevented by nec-1 (Figure 4d) or FasL neutralization (Figure 4e). Moreover, inhibition of the known downstream effector pathways of RBC necroptosis, including formation of ceramide, reactive oxygen species, and AGEs,^1^ results in inhibition of enhanced RBC death by PLY and CL-CD59 (Figures 4f and h).

### The induction of RBC necroptosis by hCD59 crosslinking and pore formation depends on the size and composition of the membrane pore

**Figure 5:**
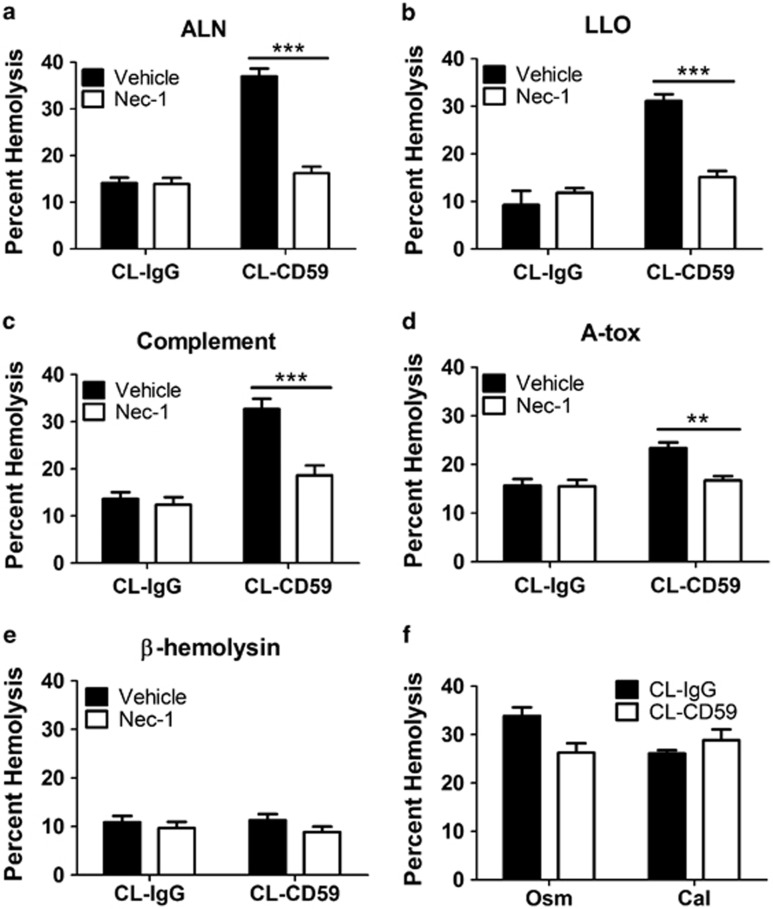
RBC necroptosis induced by hCD59 depends on membrane pore size and nature. When combined with the hCD59-independent cholesterol-dependent cytolysins (CDCs) (**a**) arcanolysin (ALN) or (**b**) listeriolysin O (LLO) or (**c**) the MAC of complement, hCD59 crosslinking (CL-CD59) induces robust RBC necroptosis as indicated by inhibition of RBC death with nec-1 (50 *μ*M). ALN, LLO, and the MAC all form protein-based pores ≥10 nm in diameter. (**d**) When combined with the hCD59-independent A-tox, which forms protein-based pores of 1–2 nm in diameter, induction of RBC necroptosis by hCD59 is weak. (**e**) When combined with *β*-hemolysin, which forms polyene-based pores, hCD59 crosslinking fails to induce RBC necroptosis. All PFTs were used at 0.2 HU. (**f**) hCD59 crosslinking does not induce RBC necroptosis when combined with two forms of eryptosis damage, hyperosmotic stress (osm) and excess calcium (cal). All hemolysis assays except for eryptosis were completed at a time point of 30 min. Eryptosis stimuli were added to RBCs over a period of 24 h. When Nec-1 was used, RBCs were treated for 1 h before the addition of stimuli. CL-IgG=crosslinked irrelevant IgG control. Error bars in hemolysis assays represent standard deviation. All hemolysis assays are the results of three independent experiments. Two-way ANOVA with Bonferroni posttest, ***P<0.001, **P<0.01

As hCD59 binding/crosslinking led to RBC necroptosis when combined with PLY, we hypothesized that hCD59 signaling might induce RBC necroptosis when combined with any agent that forms membrane pores. Therefore, we examined the effect of CL-CD59 on RBC death caused by the cholesterol-dependent cytolysins arcanolysin and listeriolysin O, the complement MAC, the cholesterol-independent PFT *S. aureus α*-toxin (A-tox), or the polyene PFT of *Streptococcus agalactiae β*-hemolysin/cytolysin (Figure 5). When CL-CD59 was combined with the cholesterol-dependent cytolysins, ALN or LLO, or the MAC it resulted in a level of RBC necroptosis similar to CL-CD59 with PLY (Figures 5a and c). However, when combined with A-tox, which forms pores of 1–2 nm in diameter ^4^ in contrast to the large pores (≥10 nm) formed by cholesterol-dependent cytolysins^9^ and the MAC,^10^ CL-CD59 induced a much lower level of necroptosis in RBCs (Figure 5d). When combined with the non-protein PFT *β*–hemolysin/cytolysin, CL-CD59 failed to induce RBC necroptosis (Figure 5e). The pore size for *β*–hemolysin/cytolysin has not been formally determined but is thought to be large.^11^ Thus, RBC necroptosis induced by CL-CD59 combined with PFTs depends on pore size and perhaps nature (protein *versus* non-protein). RBC necroptosis induced by hCD59 signaling appears specific to damage by membrane pore formation, as CL-CD59 did not enhance death via eryptosis (Figure 5f).

### Pore formation is critical for the induction of RBC necroptosis by hCD59 signaling

**Figure 6:**
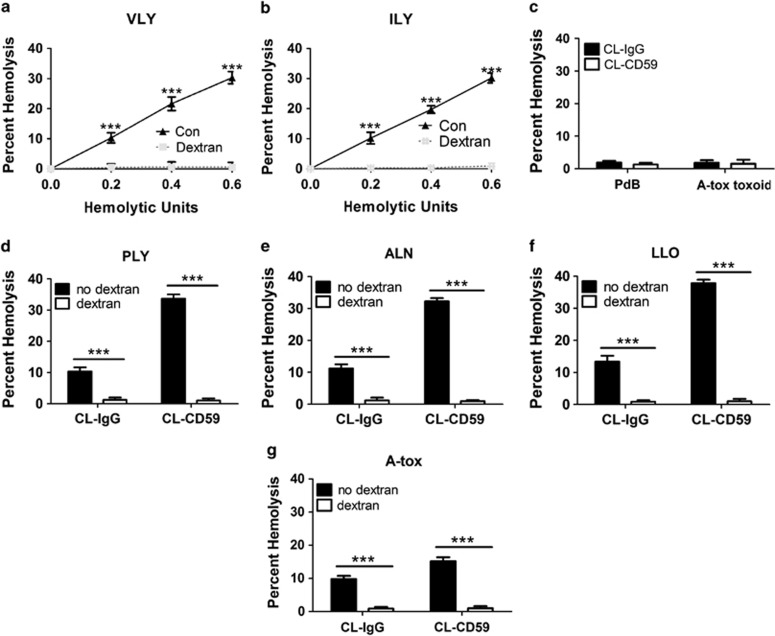
Functional pore formation is necessary for hCD59-induced RBC necroptosis. (**a** and **b**) Hemolysis assays showing that osmotic protection with dextran (500 000 MW) prevents all RBC death by the RBC necroptosis stimuli VLY and ILY. (**c**) Toxoids of PLY (PdB) and A-tox (A-tox toxoid), which are defective for pore formation, do not induce RBC necroptosis when combined with hCD59 crosslinking (CL-CD59). RBC necroptosis induced by hCD59 crosslinking combined with (**d**) PLY, (**e**) ALN, (**f**) LLO, or (**g**) A-tox is completely prevented by osmoprotection with dextran. Time points for all hemolysis assays were 30 min. PLY, ALN, LLO, and A-tox were used at 0.2 HU. CL-IgG=crosslinked irrelevant IgG control. Error bars in hemolysis assays represent standard deviation. All hemolysis assays are the results of three independent experiments. Two-way ANOVA with Bonferroni posttest, ***P<0.001

PFTs may activate host cell signaling pathways via pore-dependent or -independent mechanisms.^4^ We tested whether RBC necroptosis triggered by CL-CD59 and PFTs required functional pores. RBC death caused by the hCD59-specific PFTs, VLY and ILY, was abolished by dextran osmoprotection (Figures 6a and b). Additionally, mutant versions of PLY and A-tox that are capable of binding but are defective in membrane pore formation (PdB and A-tox toxoid, respectively)^8, 9, 10^ did not induce RBC death with or without CL-CD59 (Figure 6c). Moreover, RBC necroptosis induced by the combination of CL-CD59 with PLY, ALN, LLO, or A-tox was completely inhibited under osmoprotective conditions (Figures 6d and g). These results collectively demonstrate that functional membrane pore formation is required for hCD59-induced necroptosis in RBCs.

### RBC necroptosis is associated with echinocyte transformation

**Figure 7:**
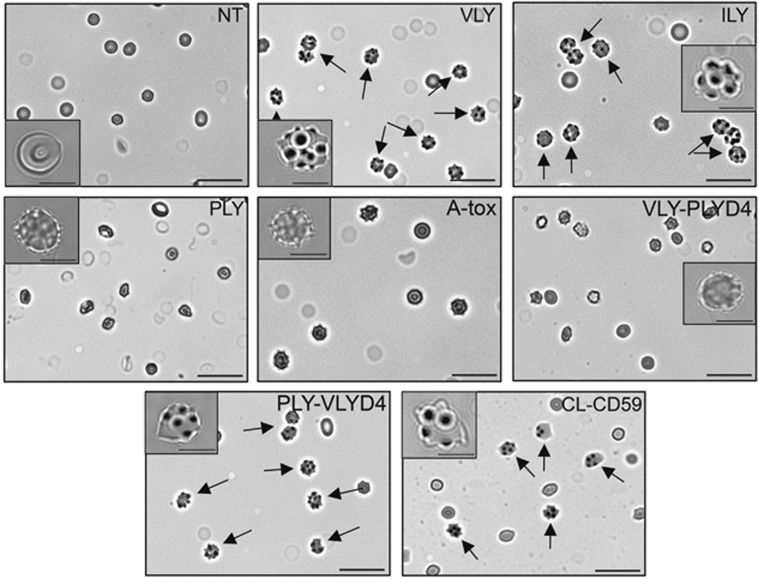
RBC necroptosis and hCD59 crosslinking are associated with the formation of echinocytes. RBCs were treated with no toxin (NT), VLY, ILY, PLY, A-tox, VLY-PLYD4, PLY-VLYD4, or with crosslinking mAbs against hCD59 (CL-CD59) and viewed by phase-contrast microscopy. RBCs transformed into the vesicluating phenotype of echinocytes in response to the RBC necroptosis stimuli VLY and ILY or CL-CD59. A chimera of PLY modified with the binding domain of VLY (PLY-VLYD4) also induced echinocyte transformation of RBCs. The hCD59-independent PFTs, PLY and A-tox, failed to induce echinocyte transformation. Additionally, a chimera of VLY modified with the binding domain of PLY (VLY-PLYD4) did not induce echinocyte transformation. Time points for all conditions were 10 min. Magnification= × 40, size bars=50 *μ*m; insets—magnification= × 100, size bars=10 *μ*m

We next examined the morphology of RBCs exposed to hCD59-specific PFTs or to mAb-based hCD59 crosslinking (Figure 7). When exposed to VLY or ILY, which induce RBC necroptosis, RBCs underwent a morphological transformation to echinocytes (Figure 7). Echinocytes are a phenotype of RBCs that actively form and release vesicles and can be induced by a number of different forms of stress.^12^ Echinocyte transformation was not observed in response to PLY and A-tox, which do not induce RBC necroptosis. The basis for echinocyte transformation of RBCs during necroptosis depended on hCD59 binding, as VLY-PLYD4 did not induce echinocyte transformation while PLY-VLYD4 did (Figure 7). hCD59 crosslinking alone was sufficient to induce echinocyte transformation (Figure 7).

### Phosphorylation of Band 3 by Syk kinase leads to FasL release, echinocyte transformation, and RBC necroptosis

**Figure 8:**
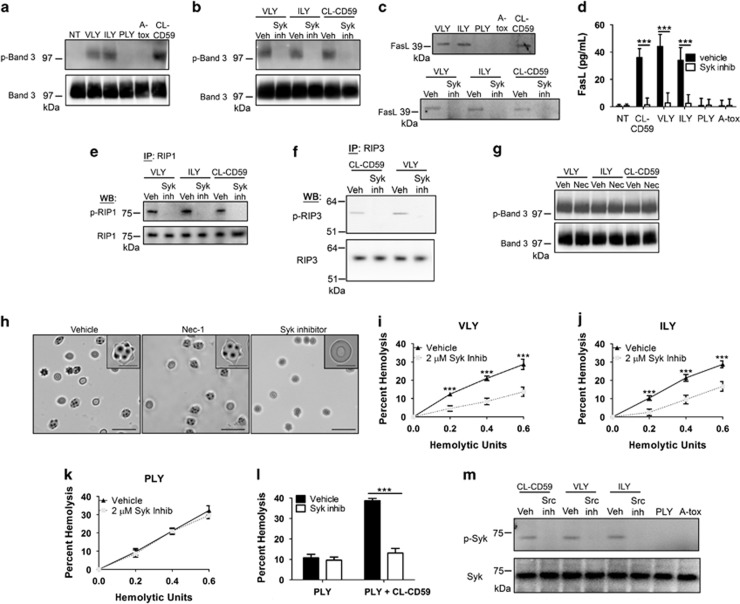
Echinocyte formation mediated by Syk-dependent Band 3 phosphorylation is necessary for FasL-dependent necroptosis in RBCs. (**a**) Western blots showing the phosphorylation of Band 3 (p-Band 3) in RBC lysates in response to the RBC necroptosis stimuli VLY and ILY or hCD59 crosslinking (CL-CD59). (**b**) The p-Band 3 induced by VLY, ILY, or CL-CD59 is prevented by inhibition of Syk kinase (Syk inh, 2 *μ*M). (**c**) Western blots of RBC supernatants showing that FasL is released from RBCs in response to sublytic doses of VLY and ILY or CL-CD59 (upper blots). The release of FasL from RBCs is prevented by inhibition of Syk kinase (Syk inh, 2 *μ*M) (lower blots). (**d**) ELISA showing that FasL is released from RBCs following treatment with sublytic doses of VLY and ILY or CL-CD59 that is prevented by inhibition of Syk kinase (Syk inhib, 2 *μ*M). The hCD59-independent PFTs, PLY and A-tox, do not induce release of FasL from RBCs. All doses of PFTs used in (**c** and **d**) are sub-lytic and did not cause any measureable RBC death. (**e**) Inhibition of Syk kinase prevents the phosphorylation of RIP1 induced by VLY, ILY, or CL-CD59 as determined by IP and western blot. (**f**) Inhibition of Syk kinase prevents the phosphorylation of RIP3 induced by VLY or CL-CD59 as determined by IP and western blot. (**g**) The phosphorylation of Band 3 induced by VLY, ILY, or CL-CD59 is not affected by the RIP1 inhibitor, nec-1 (50 *μ*M). (**h**) Induction of echinocyte formation by hCD59 crosslinking is prevented by inhibition of Syk kinase but not by inhibition of RIP1 (nec-1). Hemolysis assays showing that Syk inhibition reduce RBC death by the RBC necroptosis stimuli (**i**) VLY and (**j**) ILY but not by (**k**) the hCD59-independent PLY. (**l**) Induction of RBC necroptosis by PLY (0.2 HU) combined with hCD59 crosslinking (CL-CD59) is prevented by inhibition of Syk kinase (Syk inhib). (**m**) Western blots of RBC lysates showing that VLY, ILY, and CL-CD59 induce the phosphorylation of Syk (p-Syk), which is prevented by inhibition of Src family kinases with PP1 (Src inh, 1 *μ*M). Time points for experiments done before IPs were 1 h. Time points before microscopic analysis were 10 min. Time points for hemolysis assays were 30 min. When pharmacological inhibitors were used, RBCs were treated for 1 h before the addition of stimuli. Error bars in hemolysis assays represent standard deviation. All hemolysis assays are the results of three independent experiments. Two-way ANOVA with Bonferroni posttest, ***P<0.001

Echinocyte formation in RBCs is mediated by Syk kinase-dependent phosphorylation of the integral membrane protein Band 3.^13, 14, 15,16^ The phosphorylation of Y21 of Band 3 by Syk disrupts the binding of this protein to the adaptor protein Ankyrin, thereby severing the anchoring of the RBC membrane to the spectrin cytoskeleton.^13, 14, 15,16^ Due to this disruption, membrane vesiculation proceeds in RBCs resulting in the appearance of the echinocyte morphology.^13, 14^ As echinocyte formation was associated with RBC necroptosis (Figure 7) we asked whether Syk-dependent Band 3 phosphorylation occurred during this PCD. Indeed, phosphorylated Band 3 was observed in RBCs in response to the necroptosis-inducing hCD59-specific PFTs, VLY and ILY, but not in response to the hCD59-independent PLY and A-tox (Figure 8a). Furthermore, hCD59 signaling alone was sufficient to induce p-Band 3 as observed by mAb crosslinking of hCD59 (Figure 7a). Phosphorylation of Band 3 was also dependent on Syk, consistent with the molecular mechanism of echinocyte formation in RBCs (Figure 8b).

As Syk-dependent echinocyte formation is associated with RBC necroptosis, we considered a potential role for RBC vesiculation in this PCD pathway. FasL-dependent death in other cell types requires the release of FasL in membrane vesicles, which is followed by FasL binding to its receptor, Fas.^7, 17, 18, 19,20^ We observed FasL release from RBCs in response to sub-lytic quantities of VLY and ILY but not PLY or A-tox (Figures 8c and d). Crosslinking of hCD59 alone, which does not induce RBC death (Figure 4a), also resulted in the release of FasL from RBCs (Figures 8c and d). The release of FasL from RBCs in response to these necroptotic stimuli was dependent on Syk, suggesting that RBC vesiculation/echinocyte formation is the basis for FasL release during RBC necroptosis (Figures 8c and d). This is necessary for RBC necroptosis to proceed as the inhibition of Syk also prevented the phosphorylation of RIP1 and RIP3 induced by hCD59-specific PFTs or CL-CD59 (Figures 8e and f). As predicted, RIP1 and RIP3 phosphorylation was downstream of Syk-dependent Band 3 phosphorylation as nec-1 had no effect on this event (Figure 8g).

Syk inhibition on RBCs prevented CL-CD59-induced echinocyte transformation, but nec-1 had no effect (Figure 8h) supporting the concept that Syk-dependent Band 3 phosphorylation leads to vesiculation in RBC necroptosis and that RIP1 and RIP3 phosphorylation are downstream of this event. Syk activity was required for necroptotic death of RBCs as its inhibition resulted in a decrease of RBC death caused by VLY and ILY while having no effect on RBC death by PLY (Figures 8i and k). Inhibition of Syk also reduced RBC necroptosis caused by the combination of CL-CD59 and PLY (Figure 8l). Src family kinases interact with CD59 and phosphorylate cytosolic targets such as Syk in response to extracellular signals,^21^ which may subsequently drive vesiculation. This is indeed the case in the induction of RBC necroptosis as inhibition of Src family kinases prevented the phosphorylation of Syk induced by VLY, ILY, or CL-CD59 (Figure 8m). The hCD59-independent PFTs, PLY and A-tox, did not induce phosphorylation of Syk, consistent with the results of Band 3 phosphorylation and echinocyte formation. These results suggest that hCD59 signaling activates Src family kinases, which lead to Syk-dependent vesiculation that is required for FasL-dependent RBC necroptosis.

## Discussion

### Minimal requirements for RBC necroptosis

Our biochemical analysis of RBC necroptosis has allowed us to determine its minimal requirements. The combination of phosphorylated RIP1/RIP3 and a functional membrane pore results in necroptosis of RBCs. RIP1 phosphorylation can be induced by rFasL (Figure 1c)^1^ or by hCD59 signaling (mediated by endogenous FasL) in RBCs (Figure 2e). Additionally, hCD59 signaling leads to RIP3 phosphorylation mediated by RIP1 (Figures 3a and b). Despite the fact that these stimuli induce RIP1/RIP3 phosphorylation in RBCs they were not sufficient to cause death (Figures 1b and 4a). However, when combined with the addition of PFTs to RBCs these stimuli did induce necroptosis (Figures 1b,4d, h,5a, and d). Osmotic protection and experiments with pore-deficient toxoids (Figure 6) supported the hypothesis that the combination of phosphorylated RIP1/RIP3 and a functional membrane pore are minimally required for RBC necroptosis. Membrane pore-induced damage appears to be specifically required for RBC necroptosis, as hCD59 crosslinking had no effect on RBC death by eryptosis (Figure 5f).

Although functional pore formation is required for RBC necroptosis, the ability to stimulate necroptosis appears to depend on pore size and possibly toxin composition. hCD59 crosslinking stimulated robust necroptosis when combined with toxins that form large, protein-based pores.^22, 23^ In contrast, hCD59 crosslinking primed RBC necroptosis to a much weaker extent when combined with A-tox (Figure 5d), which forms smaller pores, and not at all with the polyene *β*-hemolysin (Figure 5e). This suggests that RBC necroptosis may be a specific response to protein-based membrane pores.

### Biochemical induction of hCD59-induced necroptosis in RBCs

**Figure 9:**
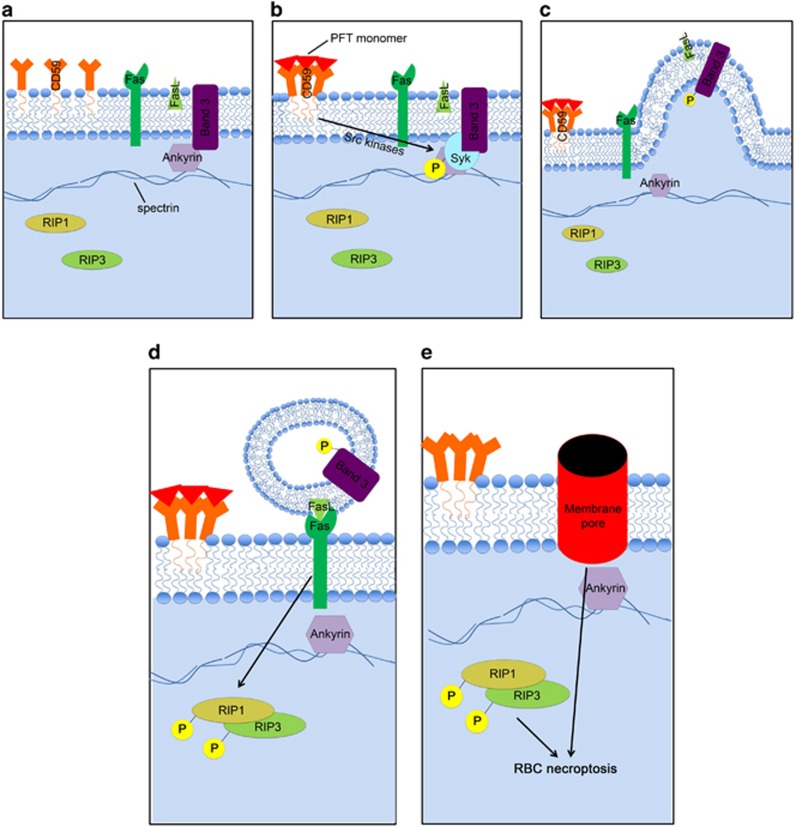
Proposed mechanism for RBC necroptosis. (**a**) RBCs contain CD59, Fas, FasL, and the transmembrane protein Band 3 in their membrane. Band 3 anchors the membrane to the spectrin cytoskeleton via the adaptor protein Ankyrin. Within the RBC cytoplasm are RIP1 and RIP3 kinases. (**b**) RBC necroptosis is intitated through CD59 crosslinking/signaling, which may be caused by the binding of hCD59-specific PFT monomers (pictured). CD59 signaling leads to the phosphorylation of Syk kinase through the action of Src family kinases. Following activation by CD59/Src kinases, Syk phosphorylates Band 3. (**c**) Syk-dependent Band 3 phosphorylation disrupts anchoring of the RBC membrane to the spectrin cytoskeleton resulting in vesiculation/echinocyte formation. (**d**) FasL is released due to Syk-dependent vesiculation/echinocyte formation and subsequently binds its cognate receptor, Fas. Fas receptor signaling, following ligation of FasL, leads to the phosphorylation of RIP1 which in turn phosphorylates RIP3, but is not sufficient to induce necroptotic death of RBCs. (**e**) Creation of a membrane pore by PFTs combined with the phosphorylation of RIP1/RIP3 leads to RBC necroptosis and the death of the RBC

Our findings have shed light on the mechanism of hCD59-induced RBC necroptosis providing a fundamental understanding of RBC necroptosis caused by hCD59-specific PFTs (Figure 9). The process of RBC necroptosis begins with ligation of the hCD59 receptor. Crosslinking of hCD59 alone leads to vesicle formation in RBCs, transforming these cells into echinocytes (Figure 7). This begins as hCD59 signaling that leads to phosphorylation/activation of Syk via Src family kinases (Figure 8m). The induction of membrane vesiculation in RBCs downstream of hCD59/Src signaling is mediated by Syk-dependent phosphorylation of the integral membrane protein, Band 3 (Figures 8a, b and 8h). The Syk-dependent formation of vesicles is the basis for the release of FasL from RBCs (Figures 8c and d). Following release from RBCs due to Syk-dependent vesiculation, FasL is free to bind its cognate receptor, Fas. The ligation of FasL with Fas leads to the phosphorylation of RIP1 (Figures 1b and 2e), which is critical for necroptosis in RBCs and other cell types.^1, 7^ It is clear that the interaction of FasL with Fas is upstream of RIP1 phosphorylation as neturalization of FasL prevents this phosphorylation downstream of hCD59 signaling (Figure 2e). Additionally, we have confirmed that FasL release is downstream of membrane vesiculation/echinocyte formation as inhibition of Syk prevents the release of FasL and the phosphorylation of RIP1 (Figures 8c and e). Moreover, FasL release occurs in the absence of RBC death, suggesting that this event occurs before the induction of necroptotic death (Figures 8c and d). The RIP1 inhibitor, nec-1, had no effect on the phosphorylation of Band 3 (Figure 8g) providing further confirmation that membrane vesiculation/echinocyte formation is upstream of RIP1 phosphorylation in RBC necroptosis. FasL-dependent RIP1 phosphorylation subsequently results in the phosphorylation of RIP3, which is required for RBC necroptosis (Figure 3). While hCD59 signaling alone causes FasL-dependent RIP1/RIP3 phosphorylation via Syk-dependent membrane vesiculation in RBCs, this is not sufficient to promote death by necroptosis (Figure 4a). Functional pore formation in the RBC membrane was shown to be necessary for necroptosis in these cells (Figure 6). This leads to the conclusion that while RIP1/RIP3 phosphorylation and functional pore formation are each necessary for RBC necroptosis they are sufficient to promote this PCD only in combination.

Although Syk-dependent membrane vesicle production/echinocyte formation is not specific to RBC necroptosis, our findings indicate that this event is critical for necroptosis to proceed. This is evident as the inhibition of Syk resulted in a decrease in RBC death caused by the necroptosis-inducing hCD59-specific PFTs, VLY and ILY while having no effect on death by PLY, which does not induce RBC necroptosis (Figures 8i and k). Further evidence of the requirement of Syk-dependent vesiculation/echinocyte formation for RBC necroptosis was shown as Syk inhibition completely prevented this PCD induced by the combination of crosslinked hCD59 and PLY (Figure 8l).

In nucleated cell types, necroptosis is mediated by one of three ligands of the TNF superfamily, TNF-*α*, FasL, or TRAIL.^5, 6, 7^ Our results suggest that in RBCs, necroptosis is mediated only by FasL. This is evident as addition of rFasL to RBCs resulted in the phosphorylation of RIP1 while addition of rTNF-*α* or rTRAIL did not (Figure 1b). Furthermore, while rFasL endows bacterial PFTs with the ability to induce RBC necroptosis regardless of intrinsic hCD59 specificity (Figure 1a), rTNF-*α* and rTRAIL do not (Figures 1d and f). It is also clear that RBC necroptosis induced by hCD59-specific PFTs is specifically mediated by endogenous FasL^1^ and not TNF-*α* or TRAIL (Figures 1c and e).

### Potential relevance of RBC necroptosis to pathological conditions

That the combination of phosphorylated RIP1/RIP3 (induced by FasL alone or hCD59 signaling) and certain PFTs results in necroptosis of RBCs (Figures 1a, 4d, h,5a, and d) may be of relevance to pathologic situations *in vivo*. In the context of infection by an organism such as *S. pneumoniae*, FasL has been shown to be present at high levels *in vivo*^24^ and *in vitro.*^25^ As infection with *S. pneumoniae* can progress to bacteremia^21^ it is possible that the combination of circulating FasL and PLY leads to RBC necroptosis as a component of systemic infection. Moreover, as the natural function of hCD59 is to prevent the formation of the MAC through binding of complement components C8 and C9, we speculate that RBC necroptosis may occur during situations of autoimmune complement attack. In a situation such as this we would expect C8/C9 to be bound to hCD59 on RBCs, eliciting signal transduction by this receptor. This combined with formation of a functional MAC may produce RBC necroptosis as is the case for CL-CD59 and the MAC (Figure 5c). This idea is supported by the evidence that hCD59 signaling induced by C8/C9 mimicks the signal transduction caused by mAb crosslinking of this receptor *in vitro.*^26^

## Materials and Methods

### Human RBCs

Primary human RBCs were obtained from healthy volunteers under a protocol approved by the Columbia University Institutional Review Board.

### Receptor crosslinking

Crosslinking of hCD59 was achieved with anti-CD59 mAb MEM-43 (1 *μ*g/ml, ThermoScientific, Waltham, MA, USA) and goat anti-mouse IgG (1 : 100, Jackson ImmunoResearch, West Grove, PA, USA). hCD59 crosslinking was also achieved with a GFP and His-tagged version of the VLY binding domain (10 *μ*g/ml, VLYD4-GFP) and mouse anti-His mAb (1 : 100, Sigma-Aldrich, St. Louis, MO, USA) or chicken anti-GFP pAb (1 : 100, EMD Millipore, Billerica, MA, USA). hCD55 crosslinking was achieved with anti-CD55 mAb (10 *μ*g/ml, ThermoScientific) and goat anti-mouse IgG (1 : 50, Jackson ImmunoResearch). As negative controls, His-tagged PLYD4 (10 *μ*g/ml) and anti-His mAb (1 : 50, Sigma-Aldrich) or rGFP and chicken anti-GFP pAb (1 : 50, EMD Millipore) was added to RBCs. For hemolysis assays, receptor crosslinking was performed concurrently with PFT treatment. For immunoprecipitations (IPs), receptor crosslinking was performed at 37 ^o^C for 30 min followed by the IP procedure.

### Pharmacological and mAb-based inhibitors

For neutralization of FasL, TNF-*α*, or TRAIL, mAbs NOK-1 (Becton DIckenson, Franklin Lakes, NJ, USA), J1D9 (Axxora, Farmingdale, NY, USA), or RIK-2 (Becton DIckenson), respectively, were used at 1 *μ*g/mL. Inhibition of RIP1 kinase was achieved with 50 *μ*M necrostatin-1 (EMD Millipore). Inhibition of RIP3 was achieved with 2 *μ*M GSK'872 (EMD Millipore). Inhibition of acid sphingomyelinase was achieved with 20 *μ*M desipramine hydrochloride (DPA, Tocris Bioscience, Bristol, UK). Iron chelation was achieved with 100 *μ*M 2,2-bipyridyl (Alfa Aesar, Ward Hill, MA, USA). Inhibition of AGE formation was achieved with 1 mM pyridoxamine (Acros Organics, Waltham, MA, USA). For inhibition of Syk kinase, Syk kinase inhibitor IV, Bay 61-3606 (EMD Millipore) was used at 2 *μ*M. Inhibition of Src family kinases was achieved with 1 *μ*M PP1 (EMDf Millipore). Osmotic protection was performed with 10% dextran (500 000 MW) in DPBS.

### Hemolysis assays

Recombinant PFTs were expressed and purified as previously described.^11, 27, 28^ *β*-Hemolysin was extracted from *S. agalactiae* according to the method of Liu *et al.*^29^ Hemolysis assays were performed in DPBS at 37 °C for 30 min with PFTs at the indicated hemolytic units (HU). 1 HU is defined as the concentration at which a PFT causes 50% hemolysis.^1^ When mAb or pharmacological inhibitors were used they were incubated with RBCs for 1 h at 37 °C before PFT addition. rFasL (Super Fas ligand, Enzo Life Sciences, Farmingdale, NY, USA), rTNF-*α* (Invitrogen, Waltham, MA, USA), or rTRAIL (Killer TRAIL, Enzo Life Sciences) was added to RBCs at the same time as PFTs. Receptor crosslinking was performed concurrently with PFT treatment. Hemolysis was measured by relative quantification of hemoglobin release in a spectrophotometer at 415 nm.

Hemolysis by the MAC was induced by the classical complement pathway. RBCs were treated with rabbit anti-RBC mAb (1 : 100, Rockland Immunochemicals, Limerick, PA, USA) for 1 h at 37  °C followed by treatment with several dilutions of rabbit complement serum (Fitzgerald, Acton, MA, USA) in DPBS.

### Immunoprecipitations

IPs for RIP1 and RIP3 were performed on RBC sonicates with 10 *μ*g anti-RIP mAb (clone G322-2, Becton DIckenson) for RIP1 or 10 *μ*g anti-RIP3 mAb (clone Rippy3, ProSci, Poway, CA, USA) at 4 °C overnight following pre-clearing. Protein G Plus Agarose beads (100 *μ*l, Pierce, Rockford, IL, USA) were then added at room temperature for 2 h. After five washes with DPBS, agarose bead immunoprecipitates were suspended in 1 × NuPAGE LDS buffer (Invitrogen), boiled for 10 min, and run on SDS-PAGE followed by western blot transfer onto PVDF membranes. All PFT treatments were conducted by treating RBCs with 0.5 HU of PFT for 30 min at 37 °C before IP procedure. VLY-PLYD4 and PLY-VLYD4 were added to RBCs at a concentration of 1 *μ*g/ml.

### Immunoblots

For phosphorylated RIP1 (p-RIP1) and RIP3 (p-RIP3) detection, blots were blocked/washed with 5% BSA in TBS. A rabbit mAb (100G7E, Cell Signaling Technology, Danvers, MA, USA) against phosphor-Ser/Thr was used at 1 : 1000 to detect p-RIP1 and p-RIP3 as previously.^1, 30^ For p-Band 3 or p-Syk detection, RBC lysates (5% (v/v)) were probed with anti-phospho Y21 Band 3 mAb (1 : 1000, AbCam, Cambridge, MA, USA) or anti-phospho Syk (Y525/526) (clone C87C1, 1 : 1000, Cell Signaling Technology) and blocked/washed with 5% BSA in TBS.

All other blots were blocked/washed with 5% milk in DPBS. Total RIP1 was detected with anti-RIP mAb (G322-2, Becton DIckenson) at 1 : 1000. Total RIP3 was detected with polyclonal anti-RIP3 (ThermoScientific) at 1 : 1000. Total Band 3 was detected with anti-Band 3 mAb (BIII-136, Sigma-Aldrich) at 1 : 1000. Total Syk was detected with polyclonal anti-Syk (Cell Signaling Technology) at 1 : 1000. FasL was detected with rabbit pAb against FasL (ab15285, AbCam) at 1 : 250. Anti-mouse or anti-rabbit secondary horseradish peroxidase conjugates (1 : 1000, ThermoScientific) were used to detect primary antibodies. Western blots were developed with Lumi-Light substrate (Roche, Nutley, NJ, USA) for 5 min at room temperature and read in a FluorChem E imager (ProteinSimple, San Jose, CA, USA).

### Phase-contrast microscopy

Human RBC suspensions (0.5% (v/v)) were treated with 0.2 HU of PFT or 1 *μ*g/ml of anti-CD59 mAb (MEM-43, ThermoScientific) and goat anti-mouse IgG (1 : 100, Jackson ImmunoResearch) for 15 min at room temperature. Unstained RBCs were viewed under bright-field using an Axio Observer.Z1 microscope (Zeiss, Thornwood, NY, USA) and an AxioCam MRm digital camera (Zeiss).

### FasL release assay

FasL release assays were performed with 10% RBC suspensions. RBCs were treated with sub-hemolytic doses of PFTs^1^ or were treated with crosslinking mAbs against hCD59 as described above for 1 h at 37 °C. RBCs were centrifuged and supernatants were run on SDS-PAGE and transferred onto PVDF for western blot analysis or were analyzed via ELISA (Fas ligand ELISA kit, AbCam).

### Eryptosis

Hyperosmotic eryptosis was induced by 24 h incubation of RBCs (1%) in DPBS containing 950 mM sucrose at 37 °C. Calcium-induced eryptosis was achieved by incubation of RBCs (1%) with 1 mM exogenous CaCl_2_ for 24 h at 37 °C.

## Abbreviations

AGEs: advanced glycation end products
ALN: arcanolysin
aSMase: acid sphingomyelinase
CDC: cholesterol-dependent cytolysin
CL-CD59: crosslinked CD59
CL-D4: crosslinked VLY domain 4
DPA: desipramine hydrochloride
FasL: Fas Ligand
GFP: green fluorescent protein
hCD59: human CD59
ILY: intermedilysin
LLO: listeriolysin O
mAb: monoclonal antibody
MAC: membrane attack complex
MLKL: mixed lineage kinase domain-like
NOX: NADPH oxidase
nec-1: necrostatin-1
pAb: polyclonal antibody
PCD: programmed cell death
PFTs: pore-forming toxins
PLY: pneumolysin
PLY-D4: PLY domain 4
p-RIP1: phosphorylated RIP1
p-RIP3: phosphorylated RIP3
p-Syk: phosphorylated Syk
RBCs: red blood cells
RIP: receptor-interacting protein kinase
ROS: reactive oxygen species
TNF: tumor necrosis factor
TRAIL: TNF-related apoptosis-inducing ligand
VLY: vaginolysin
VLY-D4: VLY domain 4
